# Supplementary material for: Genetic comparisons of interleukin-17 reveal a framework for complex signaling evolution
Source: bioRxiv. 2026 Apr 14:2026.04.13.718218. Preprint. [Version 1] doi: 10.64898/2026.04.13.718218 (PMC13105070; doi:10.64898/2026.04.13.718218)
Supplement: Supplement 3 [file media-3.zip › Supplemental files for paper/ERC/IL17xIL17_comparisons/IL17REL_IL17RB.pdf]

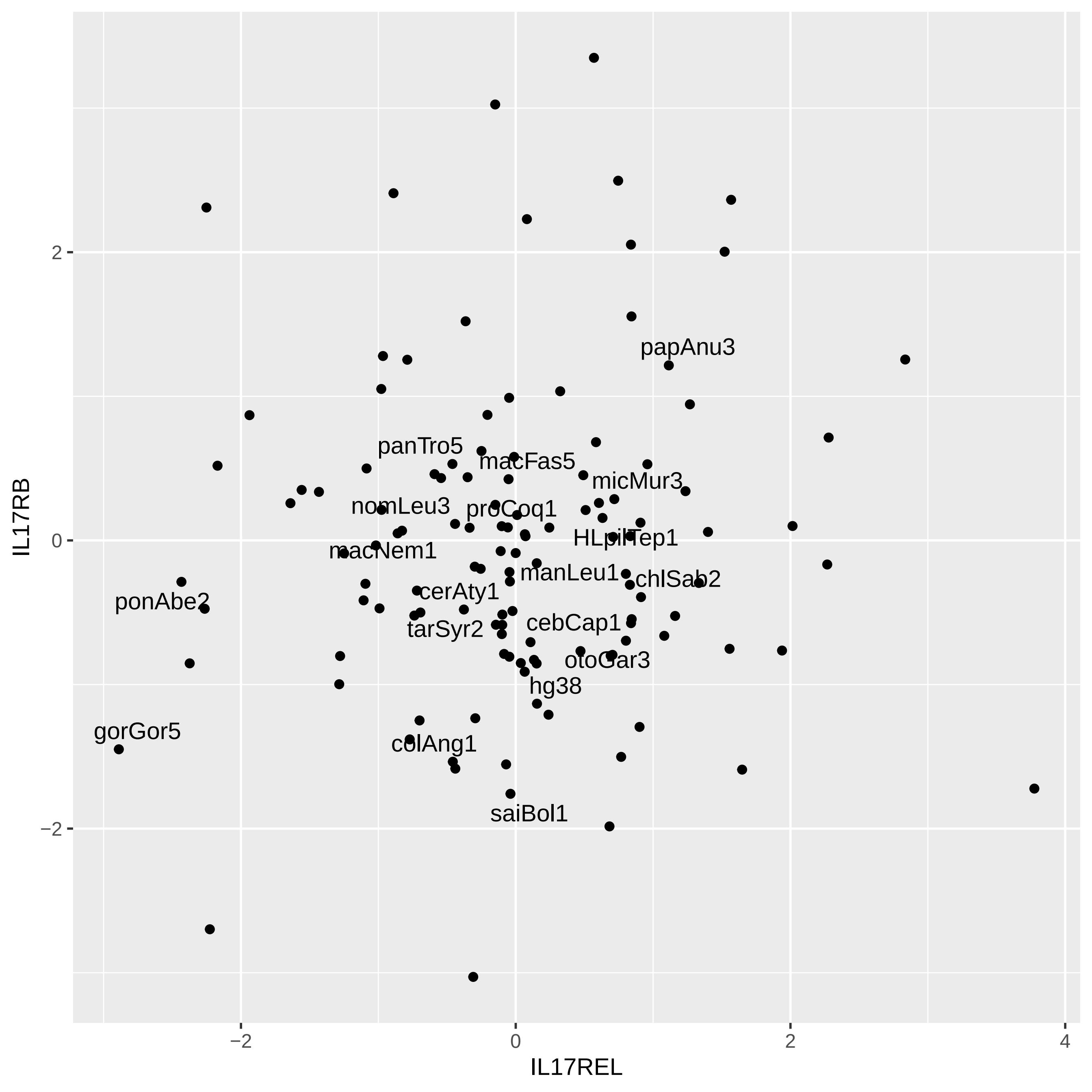

papAnu3
panTro5
macFas5
micMur3
nomLeu3
proCoq1
HLpilTep1
macNem1
manLeu1
chlSab2
cerAty1
ponAbe2
cebCap1
tarSyr2
otoGar3
hg38
gorGor5
colAng1
saiBol1
2
0
−2
−2
0
2
4
IL17RB
IL17REL
